# Blood phenylalanine fluctuation in phenylketonuric children treated by BH4 or low-phenylalanine diet from birth

**DOI:** 10.1038/s41598-023-36550-1

**Published:** 2023-06-12

**Authors:** Maurane Theron, Elise Jeannesson, Marie Canton, Farès Namour, Abderrahim Oussalah, François Feillet, Arnaud Wiedemann

**Affiliations:** 1 Pediatric Unit, Reference Center for Inborn Errors of Metabolism, University Hospital of Nancy, Nancy, France; 2 Department of Molecular Medicine, Division of Biochemistry, University Hospital of Nancy, Nancy, France; 3 INSERM UMR_S 1256, Nutrition, Genetics, and Environmental Risk Exposure (NGERE), Faculty of Medicine of Nancy, University of Lorraine, Nancy, France; 4 Pediatric Intensive Care Unit, University Hospital of Nancy, Nancy, France

**Keywords:** Molecular medicine, Prognostic markers, Metabolic disorders

## Abstract

The prognosis of phenylketonuria (PKU) is related to the quality of metabolic control all life-long. PKU treatment is based on a low-Phe diet, 6R-tetrahydrobiopterin (BH4) treatment for the BH4-responsive PKU patients or enzyme replacement therapy. Fluctuations in blood phenylalanine (Phe) concentrations may be an important determinant of intellectual outcome in patients with early and continuously treated phenylketonuria (PKU). The aim of this work is to study the fluctuation of Blood Phe in patients treated by BH4 from birth in comparison with patients treated by low-Phe diet. We conducted a retrospective study in a national reference center for PKU management. We compared mean phenylalanine blood concentration and its fluctuation in 10 BH4-responder patients (BH4R) and in 10 BH4 non-responder patients (BH4NR) treated from birth. The mean blood Phe concentration is similar between the two groups before 10 years of age (290 ± 135 (BH4R) vs. 329 ± 187 µmol/L, *p* = 0.066 (BH4NR)) while it is lower in the BH4R group after 10 years of age. (209 ± 69 vs. 579 ± 136 µmol/L, *p* = 0.0008). Blood Phe fluctuation is significantly lower in the BH4R group compared to the BH4NR group (70.2 ± 75.6 vs. 104.4 ± 111.6 µmol/L, *p* < 0.01) before 6 years of age. There are no significant differences observed on nutritional status, growth, and neuropsychological tests between the two groups. BH4 introduced in the neonatal period is associated with less blood Phe fluctuation before 6 years. Additional time and patients are required to determine if the decrease in Phe fluctuation would positively impact the long-term outcome of PKU patients.

## Introduction

Phenylketonuria (PKU) is an autosomal recessive metabolic disorder due to mutations of the phenylalanine hydroxylase gene (PAH). This hepatic enzyme is required to metabolize phenylalanine (Phe) into tyrosine^1^. If untreated, PKU leads to high blood and cerebral phenylalanine (Phe) levels^1^, which induce a severe intellectual disability and neurologic disorders^2^. Neonatal screening^3^ provides early diagnosis and allows to start the treatment in the first days of life which is essential to reach an optimal outcome^4^. The mainstay of PKU treatment is based on a strict low Phe diet completed by amino acids mixtures without Phe and low-protein food products to optimize nutritional intakes^5^. When a good metabolic control is achieved, the patients can have a normal outcome and compliance to treatment is therefore essential to obtain a good outcome^6,7^. For more than ten years, Sapropterin dihydrochloride (Kuvan®, BioMarin, CA, USA), an oral synthetic formulation of BH4, is available to treat the PKU patients who are responsive to this molecule. In these patients, BH4 restores partially or completely the PAH activity leading to a better physiological status than the low Phe diet. In Europe, sapropterin dihydrochloride could be used in patients older than 4 years since 2008 but its use was recommended in France in patients younger than 4 years in 2010^8^ before the European authorization which was obtained in 2017, for this group of patients^9^.

PKU outcome research and clinical monitoring are focused on single or average blood Phe concentrations quantifying concurrent and historical exposure of the organism to Phe, the main cause of deleterious effects in PKU^10,11^. In addition to absolute Phe exposure, variability or fluctuation of Phe levels has been claimed to have an independent negative effect on intellectual outcomes of patients^12–15^, and on white matter integrity^16^.

Variation of Phe blood concentration over time is called phenylalanine fluctuation^17^. Phe fluctuations have impact on long-term neurocognitive outcome^17,18^ and during pregnancy^19^. Feldman investigates the Phe fluctuation in patients older than 6 years old and demonstrates that the fluctuation was more reduced in patients treated by sapropterin compared to those treated by a low-Phe diet^14^. As the quality of metabolic control in the first years of life is essential to reach an optimal outcome in PKU, we studied the Phe fluctuation in patients treated by BH4 since neonatal period versus patients treated only by diet.

## Methods

### PKU patients

We conducted a retrospective study in the Reference Center for Inborn Errors of Metabolism in Nancy, France. Patients were diagnosed with PKU through the systematic neonatal screening process and the BH4 responsiveness was immediately assessed by a neonatal loading test performed immediately after screening^20^. To evaluate effects of BH4, we compared BH4 responsive patients (BH4R, n = 10) to a control group composed of BH4 non-responsive patients (BH4NR, n = 10) treated by a low protein diet. Patients of both groups were chosen to be paired according to sex, age, and period of diagnosis in order to have overall comparable management between the two groups. The number of patients in each time period decreases with time (BH4R/BH4NR: 0–3 years 10/10; 4–5 years 7/8; 6 years 6/8; 7 years 5/7; 8 years 4/5; 9–10 years 3/4; 11 years 2/3, 12–13 years 2/2; 14 years 1/1). The frequency of blood Phe measurements was done in accordance with the French recommendations8 i.e. every week until 1 year, every two weeks until 12 years and monthly after. According to European guidelines, we target a Phe blood level between 120 and 360 μmol/L [2–6 mg/dL] in child under 12 years, and between 120 and 600 μmol/L [2–10 mg/dL] after 12 years and during adulthood.

Patients with mild hyperphenylalaninemia or with inborn error of tetrahydrobiopterin metabolism (BH4 synthesis or recycling deficiencies) were excluded from the study.

### PKU patients follow-up

All patients were regularly seen in outpatient clinic by both a metabolic specialist and a specialized dietitian of our center. They were seen every month until 1 year old, then every 2 or 3 months (in function of the quality of their metabolic control) to adapt diet and/or BH4 therapy according to their blood Phe controls and growth. In case of low Phe tolerance, nutritional supplements were prescribed to reach recommended energetic and proteins intakes. In BH4R group, due to the high protein content of meat, fish or eggs, all patients have, at best, a lacto-vegetarian diet.

### Phe blood concentration and Phe fluctuation

We collected all blood Phe concentrations available in the patients’ records. Blood Phe was collected on a Guthrie card at home. We recommended to our patients to realize blood Phe test in the morning at fasting and send to the lab by post. For each patient we recorded the number of Phe blood concentrations between 120 and 360 µmol/L, lower than 120 µmol/L and higher than 360 µmol/L, per 6 months period between 0 and 12 years.

We calculated Phe fluctuation per period of 6 months. We defined Phe fluctuation as the difference between blood Phe concentration and the median Phe concentration over each 6 month-period. Phe fluctuation was expressed in µmol/L.

### Clinical outcomes

The Phe tolerance was recorded by 6 months period. Growth was assessed yearly by height, weight and body mass index expressed as standard deviation. The micronutrients status (ferritin, selenium, zinc, vitamin B12 and folate) was also evaluated yearly and a deficiency was defined as a blood level below the lower normal value for each parameter. The neurocognitive development was evaluated by neuropsychological tests according to age and the year of realization (WPPSI III or IV; WISC IV or V). Adverse events were also recorded and defined as related, possibly related or not related to the BH4 treatment.

### Statistical analysis

Statistical analysis was performed with the Statview statistic software 1992. Qualitative data were presented as frequency (%). Quantitative data were presented by median and Inter Quartile Range (IQR). We compared quantitative data between BH4R and BH4NR patients using Mann–Whitney test and Pearson’s Chi2 test (with Fisher exact test if necessary) for qualitative data. Differences were considered as significant when *P* value < 0.05.

### Ethics approval and consent to participate

The study was approved by the Research and Innovation Committee of University hospital of Nancy (France) and complies with the principles of the Declaration of Helsinki. In accordance with the national regulations on retrospective studies and considering that no intervention would be carried out in humans, it was agreed ethical committee of the University Hospital of Nancy that this study did not require informed consent from the patients.

## Results

### Patients’ description (Table 1)

**Table 1:** Population description.

Patient	Sex	Birth weight (kg)	Birth size (cm)	PAH Allele 1	PAH Allele 2	NS Phe level (µmol/L)	BH4 test performed	Phe at H0 (µmol/L)	Phe at H24 (µmol/L)	Phe decrease (%)	Delay of Phe normalization (days)
BH4R1	M	3.50	NA	c.1241A > G	IVS10-11G > A	570	Yes	702	42	94	11
BH4R2	F	3.07	46.5	c.754C > T	c.1208C > T	288	Yes	474	216	54	9
BH4R3	M	3.72	50	c.500A > T	IVS10-11G > A	216	Yes	492	108	78	9
BH4R4	M	3.42	49	c.194 T < C	c.1042C > G	390	Yes	636	204	68	15
BH4R5	F	1.46	42	c.782G > A	IVS9 + 43G > T	948	Yes	1236	378	69	13
BH4R6	M	3.70	NA	c.473G > A	c.631C > A	414	Yes	564	84	85	11
BH4R7	M	4.28	52	c.1208C > T	c.754C > T	318	Yes	276	NA*	100	6
BH4R8	F	3.20	46	c.1241A > G	c.728G > A	450	Yes	714	180	75	9
BH4R9	M	3.57	51	NA	NA	420	Yes	762	150	80	9
BH4R10	M	3.37	50	NA	NA	378	Yes	522	NA*	70	7
BH4NR1	M	NA	NA	c.1045 T > G	c.1045 T > G	522	Yes	714	660	8	30
BH4NR2	F	3.13	47	c.194 T > C	c.1066-11G > A	1080	No†	–	–	–	11
BH4NR3	M	3.40	53	c.473G > A	c.754C > T	NA	Yes	1854	2088	–13	13
BH4NR4	M	2.74	NA	Del Exon 1	IVS12 + 1G > A	1074	Yes	1986	1944	2	12
BH4NR5	F	2.77	48	c.754C > T	c.1045 T > G	660	No†	–	–	–	11
BH4NR6	M	3.84	51	NA	NA	NA	Yes	1692	1668	1	14
BH4NR7	M	3.80	52	c.782G > A	c.782G > A	768	No†	–	–	–	14
BH4NR8	F	3.98	52	c.782G > C	Del Exon 10,11,12,13	1182	Yes	1068	1302	–22	13
BH4NR9	M	3.75	50	c.1042C > G	c.1222C > T	642	Yes	1416	1260	11	13
BHNR410	M	2.64	47.5	IVS10-11G > A	c.143 T > C	510	Yes	708	696	2	15

*PAH* phenylalanine hydroxylase, *BH4* 6R-tetrahydrobiopterin, *Phe* phenylalanine, *BH4R* BH4-responsive patients, *BH4NR* BH4-nonresponsive patients.

*Patients BH4R7 and BH4R9 have siblings who are sensitive to BH4; we did not realize the BH4 test because of the same genetic mutation.

^†^BH4NR patients 2, 4 and 7 are siblings of children with PKU who were tested and found to be resistant to BH4. They were therefore not tested in the neonatal period.

10 patients treated by BH4 since neonatal period and 10 PKU patients treated by diet only were included. There were 7 males and 3 females in each group. Patient 5 in BH4R group was premature (birth at 33 weeks of amenorrhea).

The mutations of 17/20 patients are described in the Table 1. As it was expected, most of phenotype were moderate in the BH4R group while most of patients of BH4NR group exhibit a severe phgenotype. Phe levels at diagnosis were significantly higher in BH4NR-group (1285 ± 510 vs. 742 ± 415 µmol/L, *P* = 0.009). The BH4 loading test, performed in the neonatal period showed a significant decrease of blood Phe in the BH4R group while no decrease was observed in the BH4NR group (− 77% ± 13.4 [BH4R] vs. + 1.6% ± 11.8 [BH4NR] *P* < 0.001).

The normalization of blood Phe levels was faster in BH4R patients (8.4 days ± 4.33 vs. 14.6 days ± 5.9 (BH4NR), *P* = 0.008). Initially, the Phe tolerance was higher in the BH4R patients (1102 mg ± 376 [BH4R] vs. 313 mg ± 67 [BH4NR], *P* < 0.0001).

Among the 10 patients of the BH4R group, only one patient needs to count Phe intake, whereas they are 9/10 in the BH4NR group (*P* value < 0.001). About amino acid mixtures, one patient in the BH4R group has a combined treatment with BH4 + AA mixture, while 100% of patient use AA mixture in the BH4NR group (*P* value < 0.001). The adherence to the treatment was excellent in the BH4R group at all ages while in the BH4NR group, the adherence to the diet decreases after 11 years old when adolescence begins.

### Phe blood concentration (Figure 1 and Table 2)

**Figure 1:**
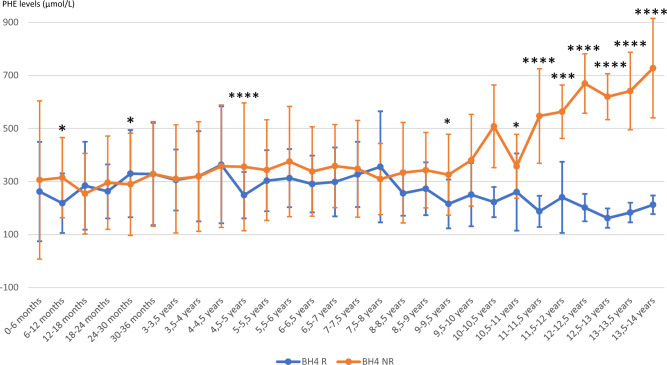
Average phenylalanine levels of BH4R and BH4NR patients over time. The values are expressed in µmol/L. The curves represent the mean levels and standard deviations of phenylalanine levels over a period of 6 months. * *P* < 0.05; ** P < 0.01; *** *P* < 0.005; **** *P* < 0.001. *Note* 20 patients were follow-up during their first of life, 16 until 2 years old, 14 until 5 years old, 10 until 6 years old, 8 until 10 years old and 4 after 10 years in each group.

**Table 2:** Number and percentage of BH4R and BH4NR levels up to three years among 3450 Phe blood concentrations.

	120–360 µmol/L			< 120 µmol/L			> 360 µmol/L		
	BH4R	BH4NR	*P* value	BH4R	BH4NR	*P* value	BH4R	BH4NR	*P* value
0–6 months n/N, % (95% CI)	**143/215, 66.6% (60.3–72.9)**	**138/245, 56.4% (50.2–62.6)**	**0.025**	32/215, 14.8% (10.1–19.5)	46/245, 18.8% (13.9–23.7)	0.267	40/215, 18.6% (13.4–23.8)	61/245, 24.9% (19.5–30.3)	0.104
6–12 months n/N, % (95% CI)	**137/164, 83.5% (78.8–89.2)**	**145/204, 71.1% (64.9–77.3)**	**0.005**	14/164, 8.5% (4.2–12.8)	31/204, 15.2% (10.3–20.1)	0.053	**13/164, 7.9%** **(3.8–12)**	**28/204, 13.7%** **(9–18.4)**	**0.079**
12–18 months n/N, % (95% CI)	**100/132, 75.8% (68.5–83.1)**	**98/167, 58.7% (51.2–66.2)**	**0.002**	**9/132, 6.8%** **(2.5–11.1)**	**34/167, 20.4% (14.3–26.5)**	** < 0.001**	23/132, 17.4% (10.9–23.9)	35/167, 21.0% (14.8–27.2)	0.44
18–24 months n/N, % (95% CI)	**84/106, 79.2% (71.5–87)**	**81/162, 50.0% (42.3–57.7)**	** < 0.0001**	**5/106, 4.7%** **(0.7–8.7)**	**32/162, 19.8% (13.7–26)**	** < 0.001**	**17/106, 16.0%** **(9.0–23.0)**	**49/162, 30.2% (23.1–37.3)**	**0.008**
24–30 months n/N, % (95% CI)	70/95, 73.7% (64.8–82.6)	80/126, 63.5% (55.1–71.9)	0.11	**1/95, 1.1%** **(0–3.2)**	**17/126, 13.5% (7.5–19.5)**	** < 0.001**	24/95, 25.3% (16.6–34)	29/126, 23.0% (15.7–30.3)	0.7
30–36 months n/N, % (95% CI)	**47/75, 62.7% (51.8–73.6)**	**62/129, 48.0% (39.4–56.7)**	**0.044**	**4/75, 5.3%** **(0.2–10.4)**	**19/129, 14.7% (8.6–20.8)**	**0.041**	24/75, 32.0% (21.4–42.6)	48/129, 37.2% (28.9–45.5)	0.45
0–36 months n/N, % (95% CI)	**581/787, 73.8% (70.7–76.9)**	**604/1033, 58.5% (55.5–61.5)**	** < 0.0001**	**65/787, 8.3%** **(6.4–10.2)**	**179/1033, 17.3% (15–19.6)**	** < 0.0001**	**141/787, 17.9% (15.2–20.6)**	**250/1033, 24.2% (21.6–26.8)**	**0.0012**

*Phe* phenylalanine, *BH4R* BH4-responsive patients, *BH4NR* BH4-nonresponsive patients.

n/N: number of Phe blood concentrations in the observed range/total number of Phe blood concentrations measured.

Significant values are in [bold].

3450 Phe blood levels were recorded during 14 years, 1287 in the BH4R group and 2163 in the BH4NR group. The median blood Phe remains in the target range for age during the first ten years in the two groups. Some statistical differences (although non clinically significant) were observed before 10 years of age; the Phe levels were higher in BH4NR patients between 6 and 12 months (314.5 µmol/L ± 151 vs. 218 µmol/L ± 112 [BH4R] *P* = 0.013) and between 4.5 and 5 years (355 µmol/L ± 241 vs. 249 µmol/L ± 88 [BH4R] *P* < 0.001) while they were lower in BH4NR patients between 24 and 30 months (289 µmol/L ± 193 vs. 330 µmol/L ± 164 [BH4R] vs. *P* = 0.033). After the age of 10 years, we observed an increase in median Phe concentrations in the BH4NR group while they remained below 300 µmol/l in the BH4R group (208 µmol/L ± 69 [BH4R] vs. 579 µmol/L ± 136 [BH4NR] *P* < 0.001).

Phe blood concentrations were more frequently observed between 120 and 360 µmol/L in BH4R patients before 3 years old (73.8 vs. 58.5% [BH4NR] *P* < 0.0001) while during the same period, Phe blood concentrations were more often higher than 360 µmol/L in the BH4NR group (24.2% vs. 17.9% [BH4R) *P* = 0.0012] or lower than 120 µmol/L (17.3% vs. 8.3% [BH4R] *P* < 0.0001).

### Phe fluctuation (Figure 2)

**Figure 2:**
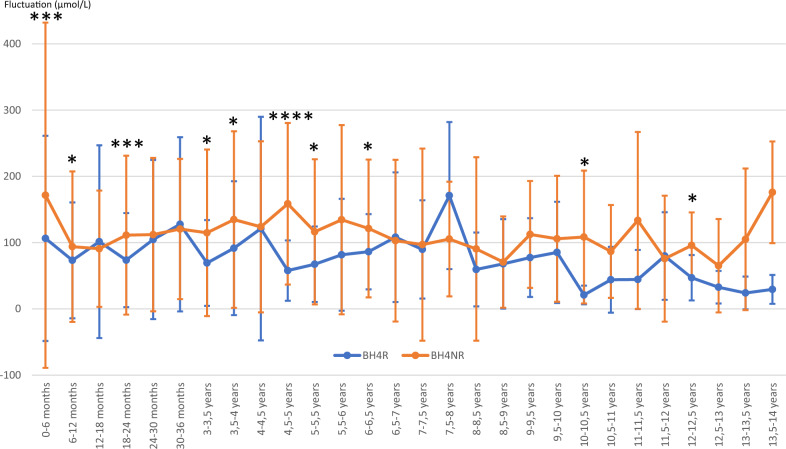
Fluctuation means of BH4R and BH4NR patients over time. The values are expressed as an average of the fluctuations in µmol/L. The curves represent the average of the phenylalanine rate fluctuations and the standard deviations over a period of 6 months. * *P* < 0.05; *** *P* < 0.005; **** *P* < 0.001. *Note* 20 patients were follow-up during their first of life, 16 until 2 years old, 14 until 5 years old, 10 until 6 years old, 8 until 10 years old, 4 after this age.

Until 6 years old Phe fluctuation was higher in the BH4NR group, except for 3 periods of 6 months: 12–18 months, 24–30 months, and 30–36 months.

There was also a significantly higher Phe fluctuation in BH4NR group for the 10–10.5 years old period (108.4 µmol/L ± 100.2 vs. 21 µmol/L ± 14.1 [BH4R] *P* = 0.005) and the 12–12.5 years old period (95.8 µmol/L ± 50 vs. 47 µmol/L ± 34.2 [BH4R] *P* = 0.04).

### Phe tolerance (Fig. 3)

**Figure 3:**
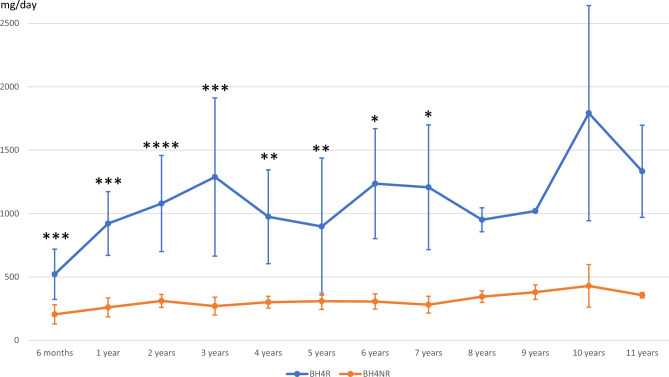
Phenylalanine tolerance of BH4R and BH4NR patients over time. Phenylalanine intake values are expressed in mg/day. * *P* < 0.05; ** *P* < 0.01; *** *P* < 0.005; **** *P* < 0.001. The absence of significance after 8 years is due to the small number of patients in these groups. *Note* 20 patients were follow-up during their first of life, 16 until 2 years old, 14 until 5 years old, 10 until 6 years old, 8 until 10 years old, 4 after this age.

The Phe tolerance rapidly increases in the first year and remains roughly higher than 1000 mg phe/day over time in the BH4R group while it did not improve in the BH4NR group (Fig. 3).

### Nutritional and growth outcomes

Anthropometric measurements at the last visit, expressed in z-score (mean +/− SD), were all in the normal range without difference between both groups (Weight: 1.36 +/− 0.43 [BHAR] vs. 2.34 +/− 0.74 [BH4NR] *p* = 0.48, Height: 1.23 +/− 0.39 [BHAR] vs. 1.17 +/− 0.370.74 [BH4NR] *p* = 0.84 and BMI: 1.28 +/− 0.4 [BHAR] vs. 1.4 +/− 0.44 [BH4NR] *p* = 0.79). Heights and weights were comparable in both groups over time. They were all normal according to national reference value. The only difference concerns BMI which was significantly lower in BH4NR patients at 6 months of age (− 0.17 ± 0.69 vs. 0.93 ± 1.06 [BH4R], *P* = 0.03).

The yearly nutritional follow up did not show any deficiency in vitamins status: no deficiency in any patient for vitamin B12 and only one low value for folates in a patient treated by BH4. Low ferritin was found in 15% of BH4R patients and in 25% of BH4NR patients. Low values of zinc and selenium were frequently observed in the two groups: 66% (BH4R) and 45% (BH4NR) for zinc 84% (BH4R) and 77% (BH4NR) for selenium. There were no significant differences in the frequency of mineral deficiency between the two groups.

### Neuropsychological tests

Neonatal BH4 therapy had no significant effect on IQ (104 ± 17 [BH4R] vs. 100 ± 16 [BH4NR] *P* = 0.5) at 6 years old in 5 BH4R patients and 4 BH4NR patients. There were not significant effects observed at three (n = 7 in each group) and eleven years old (n = 2 in each group).

### Side effects

No adverse events which could be related to BH4 treatment, were reported in treated patients.

## Discussion

It is well known that PKU patients treated by diet soon after birth have neurological outcomes close to normal^1^ and that the quality of metabolic control in the first years is crucial to optimize this outcome^3^. Despite a strict blood Phe level control, neurocognitive defects are still reported in PKU patients with early treatment^21^. Some studies questioned the relation between stability of blood Phe level and cognitive outcomes^14,22^. Fluctuation of blood Phe is defined as the variability of Phe levels over time. It is possible to estimate this fluctuation as standard deviation compared to Phe mean blood level^17^, or as the difference to median Phe blood level during a time period, like in our study. As the parents have learned to sample the Blood Phe on a Guthrie card, at fasting time in the morning before breakfast, we think that our results are not influenced by the diurnal fluctuation which has been described^23^. The day to day variation of blood Phe has also been studied and this variation can be important as van Rijn et al. showed that the blood Phe concentrations may vary up to 400% in 4 adults treated by low Phe diet^24^. This day-to-day variation can play a role in our results as we found a higher fluctuation in the BH4NR group. In 49 patients, aged from 6 to 18 years and treated by a low-Phe diet, Feldman et al. showed that the blood Phe fluctuation was higher in classical PKU patients (mean 3.3 ± 1.2 mg/dl) compared to mild PKU patients (mean 2.5 ± 0.8 mg/dl) and they hypothesized that this higher fluctuation could have a negative effect on full scale intelligence quotient^14^. On the other hand, Burton et al. showed that sapropterin therapy increased the stability of blood Phe levels and concluded that this effect is likely to improve the cognitive outcome of PKU BH4-responsive patients^25^. All these data are concordant with our results which show less fluctuation in the BH4 responsive patients treated by sapropterin from birth. Our work was made possible thanks to the neonatal BH4 loading test being performed immediately after positive neonatal screening for PKU since the early 2000s^20^. This procedure allowed us to diagnose the BH4 responsive patients before the 10th day of life and also to reduce the delay of Phe normalization in the BH4 responsive group, as we published before^20^. Our study confirms that the Phe fluctuation is less important in the moderate PKU patients treated by BH4 which is logical as these patients have a higher Phe hydroxylase activity and this enzyme activity is increased by sapropterin treatment^26^. For some of our patients who were fully responsive to BH4, the Phe tolerance can increase close to normal (up to 1800 mg/day) which means that the PAH activity normalizes and, it is logical to observe a lower Phe fluctuation with a fully restored PAH activity. Blood Phe concentration were performed according to French recommendation. In case of value outside the recommended target, changes in treatment or diet may be made at the discretion of the responsible physician. We observe a difference in the number of tests performed in the non-responsive patients (1287 in the BH4R group vs. 2163 in the BH4NR group). This higher number of Phe blood controls is observed in BH4NR patients who have a more severe phenotype related to a lower PAH activity. As the fluctuation of Phe levels is higher in this group, more blood Phe controls are needed to maintain blood Phe within the target range mainly in case of intercurrent illness.

The main question is to evaluate the impact of Phe fluctuation on the brain function. Phe concentrations can be easily measured in the blood, but brain concentrations rather than blood concentrations are considered to affect neurocognitive development^27^. Brain Phe concentration is a direct result of blood Phe concentration and Phe transport across the blood–brain barrier^28^. Such transport occurs via the neutral amino acid transporter (LAT1) and increases with Phe blood concentration, but it may also be affected by blood concentrations of other amino acids^28^. In PKU patients, the brain Phe concentration ranges from 16 to 44% of the blood concentration^16^. The variance in blood Phe concentrations (4.6%) appeared to be similar to the variance in brain values, and significant individuality of blood–brain Phe ratios is seen^29^. It has been suggested that interindividual variations in the kinetics of Phe uptake and metabolism lead to different brain Phe concentrations at comparable blood levels^30^. In summary, in patients with PKU, the increase in Phe concentrations in the brain is smaller than in the blood. Peaks in brain Phe concentrations occur later than peaks in blood concentrations, are less steep but last longer^17^.

Only a few papers, done in patients treated by diet, showed some correlations between Phe fluctuation and cognitive impairment and it is difficult to separate the effects of fluctuation and of poor metabolic control as these two parameters are both correlated to the evolution of intellectual quotient (IQ)^14,15,18,31^. A better control of Phe fluctuation may be associated with a higher IQ at adulthood^31^. Feldmann and al observed that cognitive outcome may be more related to Phe fluctuation than Phe blood level in moderate forms of PKU^14^. At the opposite, two studies failed to demonstrate a relationship between Phe fluctuation and IQ outcome^12,32^. This is why no specific statement about Phe fluctuation was included in the last PKU guidelines published in 2014 and 2017^5,33^.

Phe fluctuation from birth has never been studied, and our work is the first to describe this parameter from birth in BH4 responsive and non-responsive patients. In our study, we observed less Phe fluctuation, during the 6 first years of life, in the group of BH4-reponsive patients treated since neonatal period, whereas median Phe blood level was similar between the 2 groups. We did not demonstrate any influence of fluctuation improvement on neurocognitive outcome as the slight increase in IQ in the tested children (104 ± 17 [BH4R] vs. 100 ± 16 [BH4NR] *P* = 0.5) was not significant. Waisbren et al. reported recently that the intellectual functioning was preserved, at the end of a 7-year follow-up period, in 65 patients who began sapropterin treatment before the age of 6 years^34^ but they did not evaluate the Phe fluctuation. Therefore, it is possible that an improvement of Phe fluctuation in the first years of life could improve the neurocognitive outcome of PKU patients but larger and longer studies will be necessary to demonstrate it. Even if more than 3400 tests were performed, only 5 patients were follow-up after the age of 10 years in our cohort. Due to this low number of patients (and therefore of Phe blood tests), conclusions about this age group are limited, although there are significant differences.

In our study, BH4NR patients have more blood Phe levels below 120 µmol/L than those treated by BH4, but no one was found below 30 µmol/L which is the low-normal Phe value of our lab. It is difficult to assess the effect of Phe deficiency on a developing organism, even if this deficit is occasional like in our population. But we know that low Phe levels during pregnancy can be associated to intra uterine growth retardation^35^. Even if we do not observe that patients in the BH4NR group are smaller than in the BH4R group, a higher Phe fluctuation, with more frequent period of Phe deficiency may have an impact on children’s growth and optimal development.

Finally, considering the nutritional follow up, we report frequent micronutrients deficiencies, despite larger natural food intakes in the group treated by BH4. This result could be the consequence of a diet essentially based on vegetable proteins despite BH4 supplementation. Iron, zinc, selenium are essentially linked to animal proteins^36^ and our result shows the importance of a lack of animal protein intake in this type of deficiencies.

## Conclusion

We observed in our cohort of children treated by BH4 since the neonatal period a lower Phe fluctuation than in the group control treated only by a low Phe diet. We confirm that sapropterin produces significant and sustained reductions in blood Phe concentrations in patients with PKU responsive to BH4 and reduces fluctuations in responders. Further studies are needed to demonstrate the beneficial effect of the decrease of Phe fluctuation and to differentiate it from the direct effect of high Phe levels on neurocognitive outcome (“Supplementary information”).

## Supplementary Information

Supplementary Information.

## Data Availability

All data generated or analyzed during this study are included in this published article and its supplementary information files.

## References

[1] Blau N, van Spronsen FJ, Levy HL (2010). Phenylketonuria. Lancet.

[2] Schlegel G, Scholz R, Ullrich K, Santer R, Rune GM (2016). Phenylketonuria: Direct and indirect effects of phenylalanine. Exp. Neurol..

[3] Abadie V (2001). Neonatal screening and long-term follow-up of phenylketonuria: The French database. Early Hum. Dev..

[4] McCabe ER, McCabe L, Mosher GA, Allen RJ, Berman JL (1983). Newborn screening for phenylketonuria: Predictive validity as a function of age. Pediatrics.

[5] van Wegberg AMJ (2017). The complete European guidelines on phenylketonuria: Diagnosis and treatment. Orphanet J. Rare Dis..

[6] MacDonald A (2000). Diet and compliance in phenylketonuria. Eur. J. Pediatr..

[7] Walter JH, White FJ (2004). Blood phenylalanine control in adolescents with phenylketonuria. Int. J. Adolesc. Med. Health.

[8] Santéema, H. H. A. D. *PNDS Phénylcétonurie*. https://www.has-sante.fr/upload/docs/application/pdf/2018-06/phenylcetonurie_-_pnds.pdf (2018).

[9] Agency, E. M. *Kuvan, Summary of Product Characteristics*. https://www.ema.europa.eu/en/documents/product-information/kuvan-epar-product-information_en.pdf, https://www.ema.europa.eu/en/documents/product-information/kuvan-epar-product-information_en.pdf (2017).

[10] Huijbregts SC (2002). Sustained attention and inhibition of cognitive interference in treated phenylketonuria: Associations with concurrent and lifetime phenylalanine concentrations. Neuropsychologia.

[11] Huijbregts SC, de Sonneville LM, Licht R, van Spronsen FJ, Sergeant JA (2002). Short-term dietary interventions in children and adolescents with treated phenylketonuria: Effects on neuropsychological outcome of a well-controlled population. J. Inherit. Metab. Dis..

[12] Anastasoaie V, Kurzius L, Forbes P, Waisbren S (2008). Stability of blood phenylalanine levels and IQ in children with phenylketonuria. Mol. Genet. Metab..

[13] Arnold GL (1998). Factors affecting cognitive, motor, behavioral and executive functioning in children with phenylketonuria. Acta Paediatr..

[14] Feldmann R (2019). Children and adolescents with phenylketonuria display fluctuations in their blood phenylalanine levels. Acta Paediatr..

[15] Burgard P (1996). Phenylalanine hydroxylase genotypes, predicted residual enzyme activity and phenotypic parameters of diagnosis and treatment of phenylketonuria. Eur. J. Pediatr..

[16] Pietz J (1995). The dynamics of brain concentrations of phenylalanine and its clinical significance in patients with phenylketonuria determined by in vivo 1H magnetic resonance spectroscopy. Pediatr. Res..

[17] Cleary M (2013). Fluctuations in phenylalanine concentrations in phenylketonuria: A review of possible relationships with outcomes. Mol. Genet. Metab..

[18] Vilaseca MA (2010). Quality of dietary control in phenylketonuric patients and its relationship with general intelligence. Nutr. Hosp..

[19] Maillot F, Lilburn M, Baudin J, Morley DW, Lee PJ (2008). Factors influencing outcomes in the offspring of mothers with phenylketonuria during pregnancy: The importance of variation in maternal blood phenylalanine. Am. J. Clin. Nutr..

[20] Feillet F (2008). Evaluation of neonatal BH4 loading test in neonates screened for hyperphenylalaninemia. Early Hum. Dev..

[21] Leuzzi V, Chiarotti F, Nardecchia F, van Vliet D, van Spronsen FJ (2020). Predictability and inconsistencies of cognitive outcome in patients with phenylketonuria and personalised therapy: The challenge for the future guidelines. J. Med. Genet..

[22] Hood A, Grange DK, Christ SE, Steiner R, White DA (2014). Variability in phenylalanine control predicts IQ and executive abilities in children with phenylketonuria. Mol. Genet. Metab..

[23] Guttler F, Olesen ES, Wamberg E (1969). Diurnal variations of serum phenylalanine in phenylketonuric children on low phenylalanine diet. Am. J. Clin. Nutr..

[24] van Rijn M (2011). Adult patients with well-controlled phenylketonuria tolerate incidental additional intake of phenylalanine. Ann. Nutr. Metab..

[25] Burton BK, Bausell H, Katz R, Laduca H, Sullivan C (2010). Sapropterin therapy increases stability of blood phenylalanine levels in patients with BH4-responsive phenylketonuria (PKU). Mol. Genet. Metab..

[26] *Clinical Review Report: Sapropterin dihydrochloride (Kuvan) CADTH Common Drug Reviews* (2017). 30462435

[27] Surtees R, Blau N (2000). The neurochemistry of phenylketonuria. Eur. J. Pediatr..

[28] Moats RA, Moseley KD, Koch R, Nelson M (2003). Brain phenylalanine concentrations in phenylketonuria: Research and treatment of adults. Pediatrics.

[29] Kreis R, Zwygart K, Boesch C, Nuoffer JM (2009). Reproducibility of cerebral phenylalanine levels in patients with phenylketonuria determined by 1H-MR spectroscopy. Magn. Reson. Med..

[30] Weglage J, Wiedermann D, Moller H, Ullrich K (1998). Pathogenesis of different clinical outcomes in spite of identical genotypes and comparable blood phenylalanine concentrations in phenylketonurics. J. Inherit. Metab. Dis..

[31] Feldmann R (2019). Neurocognitive functioning in adults with phenylketonuria: Report of a 10-year follow-up. Mol. Genet. Metab..

[32] Viau KS (2011). Correlation of age-specific phenylalanine levels with intellectual outcome in patients with phenylketonuria. J. Inherit. Metab. Dis..

[33] Camp KM (2014). Phenylketonuria Scientific Review Conference: State of the science and future research needs. Mol. Genet. Metab..

[34] Waisbren S (2021). Long-term preservation of intellectual functioning in sapropterin-treated infants and young children with phenylketonuria: A seven-year analysis. Mol. Genet. Metab..

[35] Teissier R (2012). Maternal phenylketonuria: Low phenylalaninemia might increase the risk of intra uterine growth retardation. J. Inherit. Metab. Dis..

[36] Salter AM (2018). The effects of meat consumption on global health. Rev. Sci. Tech..

